# Lymphocytic Microparticles Modulate Angiogenic Properties of Macrophages in Laser-induced Choroidal Neovascularization

**DOI:** 10.1038/srep37391

**Published:** 2016-11-22

**Authors:** Houda Tahiri, Samy Omri, Chun Yang, François Duhamel, Suzanne Samarani, Ali Ahmad, Mark Vezina, Martin Bussières, Elvire Vaucher, Przemyslaw Sapieha, Gilles Hickson, Karim Hammamji, Réjean Lapointe, Francis Rodier, Sophie Tremblay, Isabelle Royal, Jean-François Cailhier, Sylvain Chemtob, Pierre Hardy

**Affiliations:** 1 Department of Pharmacology, Université de Montréal, Montréal, QC, Canada; 2 Research Center CHU Sainte-Justine, Université de Montréal, Montréal, QC, Canada; 3 Research Center Hôpital Maisonneuve-Rosemont, Université de Montréal, Montréal, QC, Canada; 4 Departments of Microbiology and Immunology, Université de Montréal, Montréal, QC, Canada; 5 Charles River Laboratories, Senneville, Montreal, QC, Canada; 6 V&O Services, Saint-Lazare, QC, Canada; 7 School of Optometry, Université de Montréal, Montréal, QC, Canada; 8 Department of Ophthalmology, Université de Montréal, Montréal, QC, Canada; 9 Department of Pathology and Cell Biology, Université de Montréal, Montréal, QC, Canada; 10 Institut du Cancer de Montréal, CRCHUM–Centre de Recherche du Centre Hospitalier de l’Université de Montréal and Department of Medicine, Université de Montréal, Montréal, QC, Canada; 11 Department of Radiology, Radio-Oncology and Nuclear Medicine, Université de Montréal, Montréal, QC, Canada; 12 University of British Columbia, Vancouver, BC, Canada; 13 Centre for Molecular Medicine and Therapeutics, Vancouver, BC, Canada; 14 Department of Pediatrics, Université de Montréal, Montréal, QC, Canada

## Abstract

Pathological choroidal neovascularization (CNV) is the common cause of vision loss in patients with age-related macular degeneration (AMD). Macrophages possess potential angiogenic function in CNV. We have demonstrated that human T lymphocyte-derived microparticles (LMPs) exert a potent antiangiogenic effect in several pathological neovascularization models. In this study, we investigated the alteration of proangiogenic properties of macrophages by LMPs treatment *in vitro* and *in vivo* models. LMPs regulated the expression of several angiogenesis-related factors in macrophages and consequently stimulated their antiangiogenic effects evidenced by the suppression of the proliferation of human retinal endothelial cells in co-culture experiments. The involvement of CD36 receptor in LMPs uptake by macrophages was demonstrated by *in vitro* assays and by immunostaining of choroidal flat mounts. In addition, *ex vivo* experiments showed that CD36 mediates the antiangiogenic effect of LMPs in murine and human choroidal explants. Furthermore, intravitreal injection of LMPs in the mouse model of laser-induced CNV significantly suppressed CNV in CD36 dependent manner. The results of this study suggested an ability of LMPs to alter the gene expression pattern of angiogenesis-related factors in macrophages, which provide important information for a new therapeutic approach for efficiently interfering with both vascular and extravascular components of CNV.

Lymphocyte-derived microparticles (LMPs) are small membrane microvesicles released from human T lymphocytes during apoptosis^1^,^2^. We have established that LMPs exert strong antiangiogenic effect *in vivo* on corneal neovascularisation, tumor neovascularization^3^,^4^, and limit neovascularization during the vasoproliferative phase of ischemic retinopathy^5^. *Ex vivo*, LMPs suppress microvessel sprouting in aortic ring^3^ and choroidal explants^6^. These effects depended upon on the integrity of the retinal pigment epithelium (RPE) and involved release of pigment epithelium-derived growth factor (PEDF) and p75 neurotrophin receptor (p75NTR)^6^. However, the role of LMPs in choroidal neovascularization (CNV) secondary to age-related macular degeneration (AMD) remains unknown.

Age-related macular degeneration (AMD) is a major cause of legal blindness in older patients industrialized countries^7^. The neovascular from of AMD is characterized by the formation of subretinal choroidal neovascularization, and it critically depends on local production of vascular endothelial growth factor (VEGF). Anti-VEGF therapy is currently the new treatment used for treating CNV, but it is plagued by unwanted side effects and/or insurmountable complications^8^. Therefore, there is an urgent need for identifying alternative approaches to treat CNV. Recent studies have suggested that immune vascular interactions play an important role in regulating angiogenesis^9^. In mouse models of AMD, macrophages and microglia (tissue-resident macrophages), recruited to the subretinal space, play a crucial role in choroidal blood vessel growth^10^,^11^. Blocking VEGF signaling inhibits the infiltration of macrophages and microglia in the murine laser-induced photocoagulation model^11^. Moreover, activation of the interferon-β signaling pathway in retinal macrophages and microglia cells reduces the inflammation and limits the development of CNV lesions^12^. These data suggest that modulation of macrophages and microglia activity may become an attractive therapeutic tool for the treatment of neovascular AMD.

Recent studies have shown that macrophages comprise an extremely heterogeneous lineage, displaying a combination of antiangiogenic and proangiogenic functions^13^. In response to microenvironmental signals, macrophages may undergo classical M1 activation (antiangiogenic) or, alternatively, M2 activation (proangiogenic)^14^. Macrophages express a panoply of cell surface receptors, including scavenger receptors such as CD36^15^, low-density lipoprotein receptors (LDLr)^16^, phosphatidylserine receptors and mannose receptors^17^. The expression of different scavenger receptors in polarized cells contributes to various pathologies.

CD36 is a transmembrane receptor that mediates diverse physiological as well as pathological processes, such as metabolism, angiogenesis and atherosclerosis^18^. Besides being expressed in macrophages, CD36 is also expressed on many other mammalian cell types including microvascular endothelial, microglial and retinal pigment epithelial cells^19^. We demonstrated previously that LMPs upregulate CD36 protein levels on endothelial cells^3^. Given the important role of CD36 in macrophages, we investigated whether LMPs modulate the angiogenic microenvironment by altering macrophage phenotype, and whether CD36 is involved in the LMPs-induced antiangiogenic effects.

## Results

### LMPs dose-dependently inhibit macrophage cell proliferation

**Figure 1:**
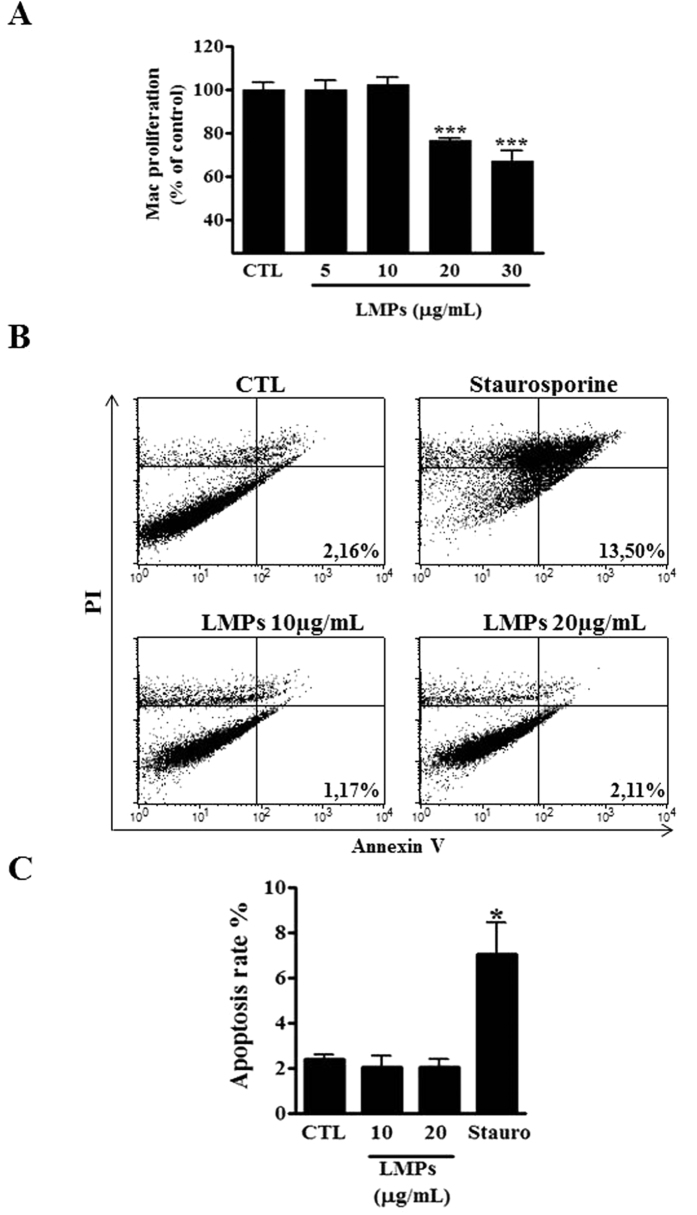
LMPs dose-dependently inhibited macrophages proliferation. (**A**) Indicated concentrations of LMPs were incubated with macrophages (RAW 264.7) for 24 hours. The proliferation of macrophages was determined using ^3^H-thymidine incorporation and values were presented as a percentage of control (CTL). ***P < 0.001 vs. CTL. (**B**) Representative results of flow cytometry analysis of macrophage cell apoptosis after 24-hours of treatment with indicated concentrations of LMPs, or staurosporine (positive control). FACS analysis was performed after macrophages were stained with Annexin-FITC and propidium iodide using Vybrant Apoptosis assay kit. Note that the staurosporin-treated cells undergo apoptosis and become PI+/Annexin V+ in the later stages of apoptosis. No difference is observed in LMPs-treated and control macrophages for Annexin V staining. **(C)** The apoptosis rates were presented as the percentage of apoptotic cells relative to the total number of cells. Values are means ± SEM. *P < 0.05 vs. CTL. Mac and Stauro indicate macrophages and staurosporin, respectively.

We have previously reported that LMPs exert not only a strong inhibitory effect on proliferation of endothelial and cancer cells^3^,^20^, but also have a pro-apoptotic effect on certain types of cells^6^,^20^,^21^. Here we observed that LMPs significantly reduced the proliferation of macrophages (RAW 264.7) in a dose-dependent manner (Fig. 1A). The FACS analysis further revealed that concentrations of LMPs below 20 μg/ml did not significantly induce macrophage cell death, although these LMPs doses significantly suppressed cell growth (Fig. 1B,C, P > 0.05). Similar results were generated by using pan caspase (FAM-VAD-FMK) fluorescent green probe for detecting active caspases in the LMPs-treated macrophages (data not shown). Thus, we chose the concentration of LMPs at 10 μg/ml for the following *in vitro* studies. Similar effects of LMPs were observed on bone marrow-derived macrophages (BMDM) from femurs of C57BL/6 mice and on human macrophages differentiated from HL60 cells (Supplementary Figures S1 and S2). Thus, we chose the concentration of LMPs at 10 μg/ml for the following *in vitro* studies.

### LMPs modulate polarization of macrophages

**Figure 2:**
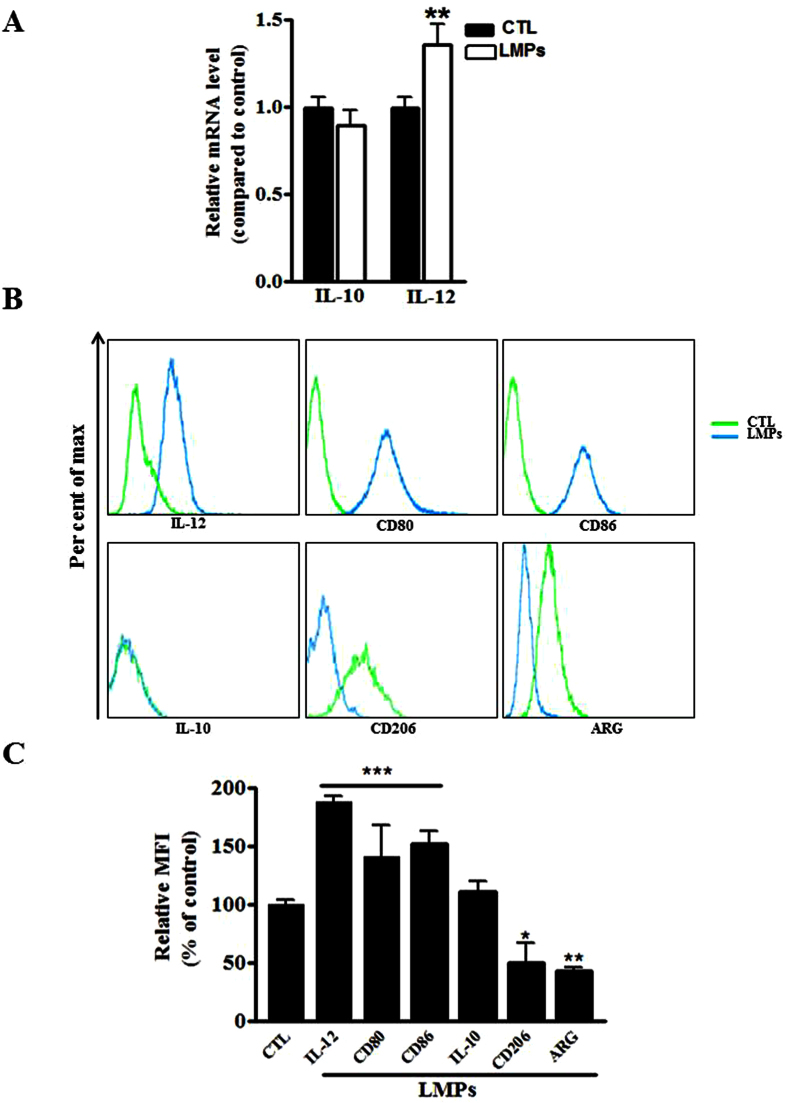
LMPs altered the expression of M1 and M2 markers of macrophages. (**A**) The mRNA expression levels of IL-10 and IL-12 were quantified by quantitative RT-PCR after macrophages (RAW 264.7) were treated with 10 μg/mL of LMPs for 24 hours. The values were presented as fold changes relative to the control group (CTL) set as 1. **P < 0.01 vs. CTL (**B**) Representative FACS analysis of the expression of IL-10, IL-12, CD80, Cd86, ARG, and CD206 in macrophages after 24-hour treatment with 10 μg/mL of LMPs. (**C**) The numbers of cells expressing of IL-10, IL-12, CD80, CD86, ARG, or CD206 were calculated respectively and presented as a percentage of CTL (set as 100%). ***P < 0.001, **P < 0.01, *P < 0.05 vs. CTL.

Macrophages have the ability to adapt to their microenvironment by undergoing phenotypic and functional changes. They have been broadly characterized by their polarization state according to the M1/M2 classification system^22^. M1 macrophages produce high levels of IL-12, whereas M2 macrophages produce high levels of IL-10. The ratio of IL-12 to IL-10 production has been effectively used to distinguish M1 and M2 macrophages^23^. To determine whether LMPs modulate the polarization of mice macrophages (RAW264.7), we first quantified the mRNA levels of IL-10 and IL-12 in macrophages after 24 hours of LMPs treatment. LMPs significantly increased the mRNA level of IL-12 in macrophages; however, they had no effect on the expression of IL-10 (Fig. 2A). In order to confirm the effects of LMPs, we performed flow cytometric analysis to detect several other specific markers for M1 or M2 macrophages. In addition to IL-12, CD80 and CD86 also serve as biomarkers for M1 macrophages^24^,^25^ while the mannose receptor C type 1 (CD206) and Arginase-1 (ARG) are commonly used as biomarkers for M2 macrophages^22^, as for IL-10. LMPs treatment significantly increased the number of macrophages expressing IL-12, CD80, or CD86 while it reduced significantly the numbers of macrophages expressing CD206, or ARG (Fig. 2B,C). Taken together, these data suggest that LMPs drive macrophages into M1 type.

### LMPs regulate angiogenic factors expression in macrophages

**Figure 3:**
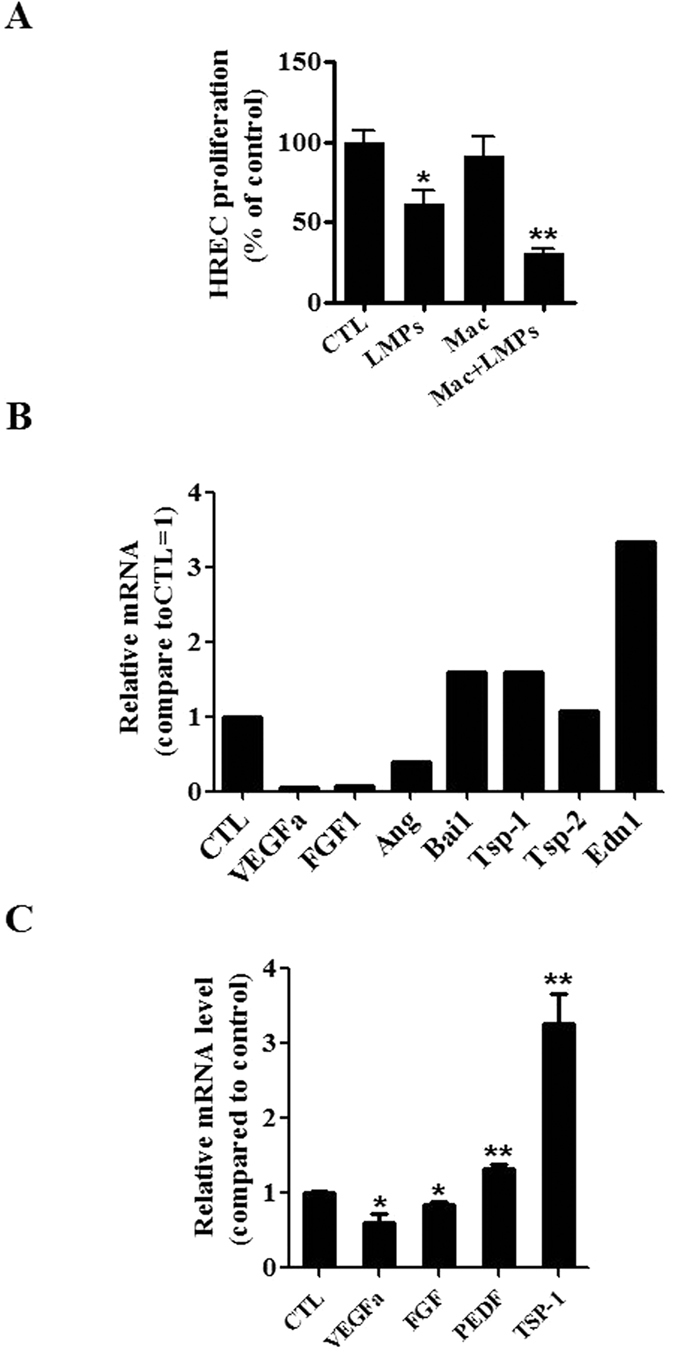
LMPs modulated angiogenesis-related factor expression in macrophages. (**A**) Cell proliferation of human retinal microvascular endothelial cells (HREC) was assessed after cells were co-cultured for 24 hours with LMPs, control medium (CTL), macrophages (RAW 264.7 pre-treated with the control medium, Mac), or LMPs pre-treated macrophages (Mac + LMPs). The relative proliferation rates of HREC were presented as a percentage of CTL. *P < 0.05, **P < 0.01 vs. CTL. (**B**) The mRNA levels of a panel of angiogenesis-related factors in macrophages (RAW 264.7) were evaluated by using a commercial PCR array after treatment with LMPs (10 μg/mL) for 24 hours. The relative mRNA level of each factor was calculated and expressed as fold change compared to control (CTL). (**C**) The gene expression of VEGFa, FGF, PEDF and TSP-1 in LMPs-treated macrophages was quantified by real-time PCR. The relative mRNA levels are presented as fold change relative to control macrophages (CTL). *P < 0.05, **P < 0.01 vs. CTL.

Macrophages possess potential angiogenic functions. They are capable of releasing angiogenic cytokines (e.g. VEGF) and are known to contribute to inflammatory neovascularization, tumor angiogenesis and choroidal neovascularization^26^,^27^,^28^. To ascertain the LMPs-induced antiangiogenic activity of macrophages, proliferation of human retinal endothelial cells (HREC) was assessed after cells were co-cultured with macrophages or LMPs-pretreated macrophages. As shown in Fig. 3A, the proliferation rate of HREC was significantly decreased by LMPs-pretreated macrophages (approximately by 65%), whereas macrophages alone had non-significant effects on endothelial cell proliferation. To answer whether LMPs directly modulate the expression of angiogenesis-related factors in macrophages, we performed a gene expression analysis to define temporal changes in a panel of angiogenesis-related factors in LMPs-treated macrophages (Supplemental Table 1). LMPs downregulated the expression of proangiogenic factors such as VEGFa, insulin-like growth factor (IGF), fibroblast growth factor (FGF) etc., but upregulated the expression of brain specific angiogenesis inhibitor 1 (Bai1), thrombospondin-1 (TSP-1), the antiangiogenic factors (Fig. 3B). We further performed the quantitative RT-PCR to confirm the expression of key angiogenic factors (highly expressed) in the choroidal tissue^29^. LMPs significantly reduced the expression of VEGFa, FGF but increased pigment *epithelium-derived factor (*PEDF) and TSP-1 expression (Fig. 3C).

### Blockade of CD36 decreases LMPs uptake by macrophages and abrogates the effect of LMPs on gene expression in macrophages

**Figure 4:**
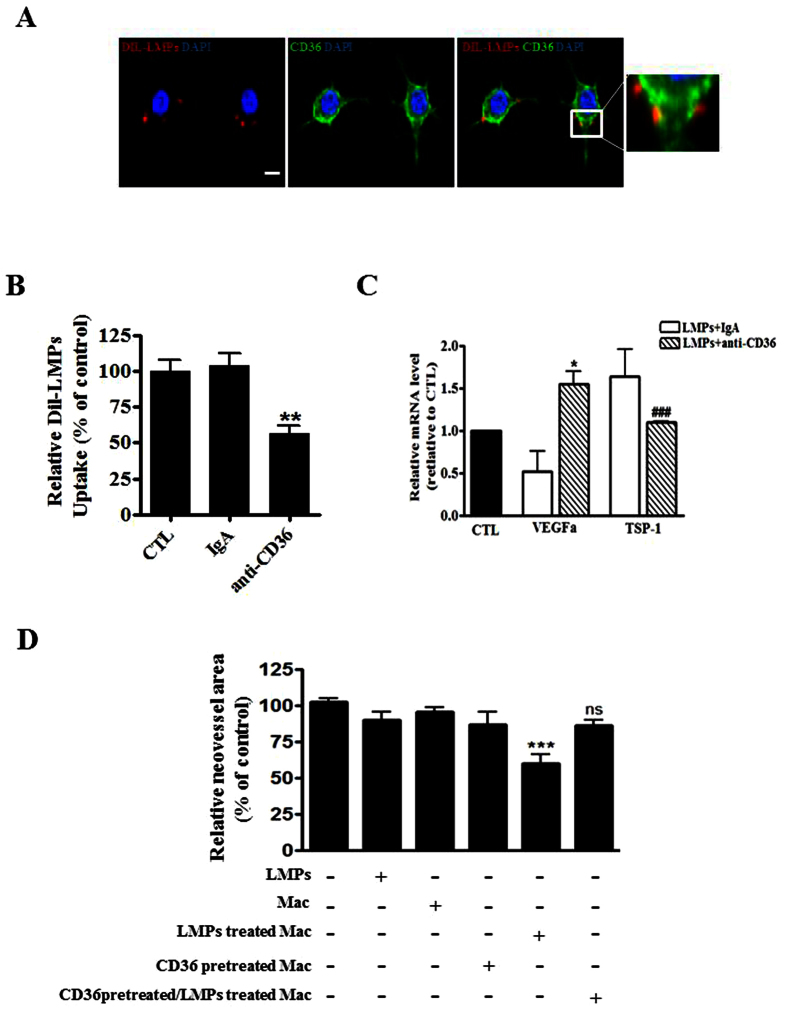
CD36 was involved in the uptake of LMPs by macrophages and mediated the effect of LMPs. (**A**) Representative images of immunohistochemistry staining of CD36 (in green) and localization of LMPs in macrophages (RAW 264.7) after 4 hours of incubation with DiI-LMPs (in red). Bar: 25 μm. The merged images on the right indicate the co-localization of CD36 and LMPs. (**B**) After a 4 h incubation of DiI-LMPs (10 μg/ml) with macrophages (control), the isotype-matched control antibody pre-treated (IgA) macrophages, or anti-CD36 antibody pre-treated macrophages, the uptake of DiI-LMPs was measured and expressed as percentage of control (CTL, set as 100%). **P < 0.01 anti-CD36 vs. CTL. (**C**) RAW 264.7 were pre-treated with control antibody (IgA) or anti-CD36 before 24 hours LMPs treatment. The mRNA levels of VEGFa and TSP-1 in the macrophages were determined by quantitative PCR. *P < 0.05, ^*###*^*P* < 0.001 vs. LMPs + IgA. (**D**) The RPE-free choroidal explants were cultured for developing new vessels in the first 5 days, and then explants were incubated for another 48 hours with 50 μg/mL of LMPs, macrophages RAW 264.7(Mac), LMPs-treated Mac, anti-CD36 pre-treated Mac or anti-CD36 pre-treated + LMPs-treated Mac. The neovascularized areas in each condition were measured and presented as a percentage of control (explants without any treatment set as 100%). ***P < 0.001 vs. CTL.

We have shown that LMPs are taken up by endothelial and epithelial cells via receptor-mediated endocytosis and phagocytosis, respectively^5^,^21^. Moreover, it is known that the membrane scavenger receptor CD36 proteins are highly expressed on macrophages membrane^30^, here we observed that CD36 is co-localized with LMPs when macrophages were incubated with DiI-LMPs (Fig. 4A). Blockade of CD36 on macrophages with anti-CD36 antibody significantly prevented LMPs uptake (Fig. 4B), suggesting that uptake of LMPs into macrophages largely depends on scavenger receptor CD36. The inhibition of CD36 consequently abrogated the effect of LMPs on VEGFa and TSP-1 expression in macrophages (Fig. 4C). Moreover, LMPs-treated macrophages exhibited strong antiangiogenic effects in the choroidal explant experiments in which the neovascularization of the RPE-free choroidal explants was decreased by 40%. This LMPs-provoked antiangiogenic effect of macrophages was attenuated by the pre-treatment of CD36 –specific antibody (Fig. 4D).

### LMPs suppress laser-induced choroidal neovascularization (CNV) *in vivo*

**Figure 5:**
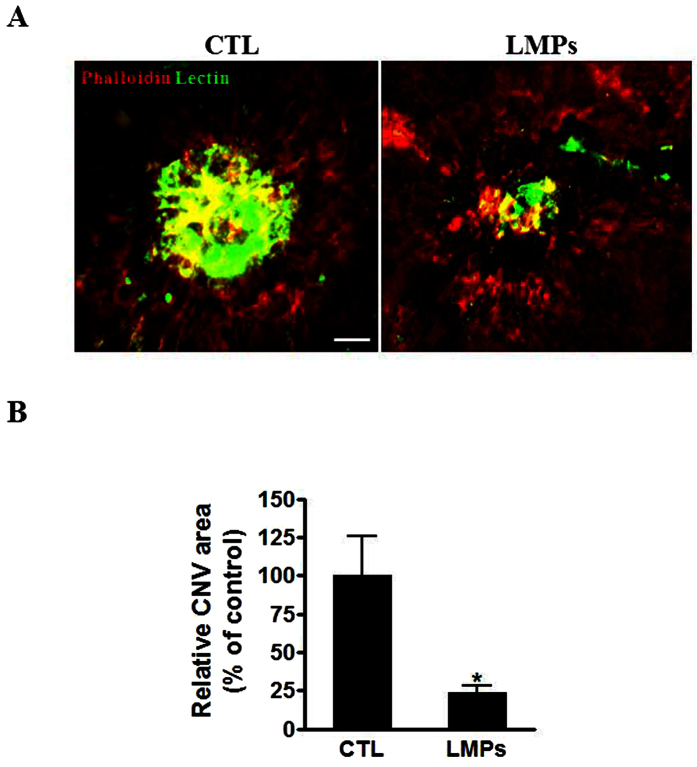
LMPs suppressed laser-induced CNV *in vivo*. (**A**) Representative images of laser-induced CNV on choroidal flat-mounts 7 days after the second intravitreal injection of 50 μg/mL of LMPs. The choroidal flat-mounts were stained with FITC-lectin (marker of endothelial cells, green) and Phalloidin (red). CTL represent the control mice that received intravitreal injections of the control medium. (**B**) CNV areas were quantified using computer-assisted semi-quantitative assay and normalized to control. Bar: 50 μm, *P < 0.05 vs. CTL. Values are means ± SEM from 10 eyes (each eye contains 3 impact laser-induced CNV) for each condition.

We have demonstrated that LMPs exert a strong antiangiogenic effect on several *in vivo* neovascularisation animal models^3^,^4^,^5^. We extended our investigation of the *in vivo* effect of LMPs using a laser-induced CNV mouse model. Following intravitreal injections of LMPs, we observed a significant decrease of the choroidal neovascularization areas (Fig. 5).

### LMPs modulate angiogenesis-related gene expression in macrophages *in vivo*

**Figure 6:**
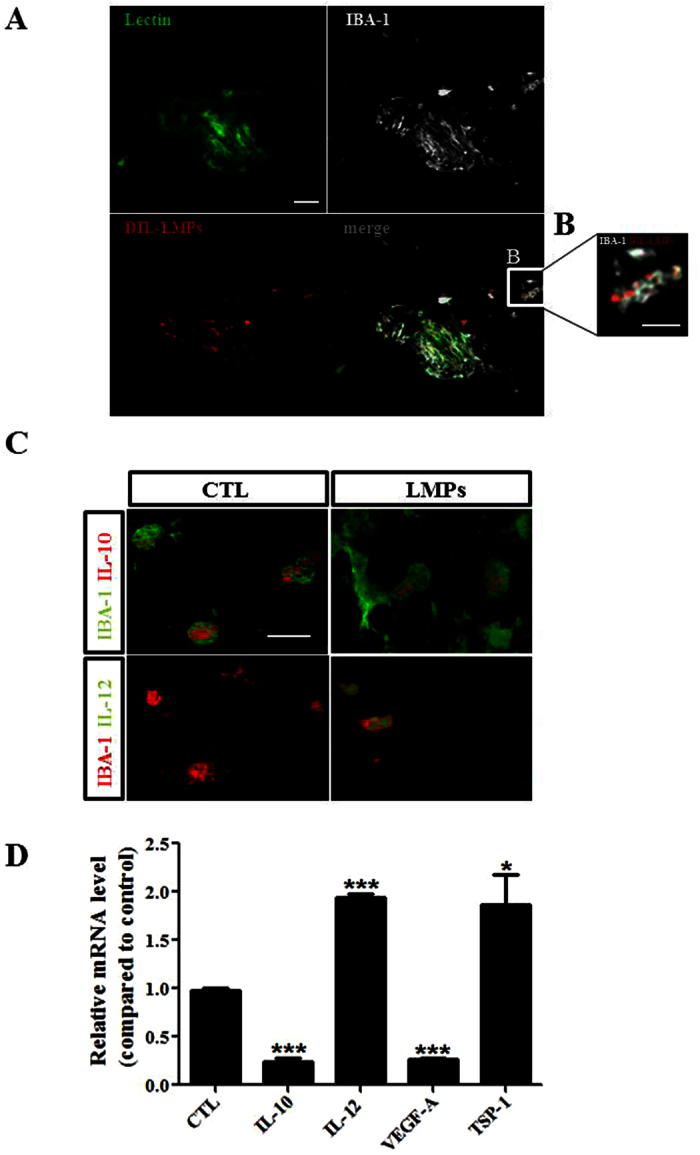
LMPs modulated gene expression in macrophages *in vivo*. (**A,B**) Representative images of LMPs uptake by macrophages *in vivo*. Immunofluorescence staining of mouse choroidal flat-mounts 7 days after the second intravitreal injections of DiI-LMPs (red) in laser-induced CNV model. The vessels were stained with FITC-lectin (green) and macrophage with IBA-1 in grey. Bar: 50 μm in (**A**) and 25 μm in (**B**). (**C**) Representative images of expression of IL-12 (green) and IL-10 (red) in macrophages in CNV areas after LMPs treatments. Bar: 15 μm. CTL represent the control mice received intravitreal injections of control medium. (**D**) Relative mRNA expression level of IL-10, IL-12, VEGFa and TSP-1 in CNV areas. Tissues from the CNV areas were collected by laser-capture microdissection. The mRNA levels of the genes of interest were quantified by quantitative RT-PCR. The values were presented as mean fold changes relative to the control (CTL) values (set as 1). *P < 0.05, ***P < 0.001 vs. CTL.

Emerging data support that macrophages contribute to pathological neovascularization^11^,^31^,^32^. To verify whether modulation of the angiogenic activity of macrophages contributes to the LMPs-mediated reduction of CNV, we first investigated the uptake of LMPs by macrophages *in vivo* (Fig. 6A,B). Fluorescent confocal microscopy revealed the presence of macrophages (IBA-1^+^ cells) in the CNV areas (Lectin-positive, in green), and LMPs co-localized with retinal macrophages after intravitreal injections of Dil-LMPs (in red) (Fig. 6B). Immunofluorescence staining also shown the expression of IL-10 and IL-12 in macrophages in the CNV areas, and LMPs induced expression of IL-12 in macrophages (Fig. 6C). To have a better understanding of the expression pattern of angiogenesis-related factors involved in this process, laser capture microdissection was performed to collect tissues in the CNV regions, the key factors were analyzed by quantitative PCR. Consistent with *in vitro* studies, LMPs significantly induced expression of IL-12 and TSP-1 but decreased the expression of VEGFa and IL-10 (Fig. 6D).

### Antiangiogenic effect of LMPs is attenuated in CD36 Knockout mice

**Figure 7:**
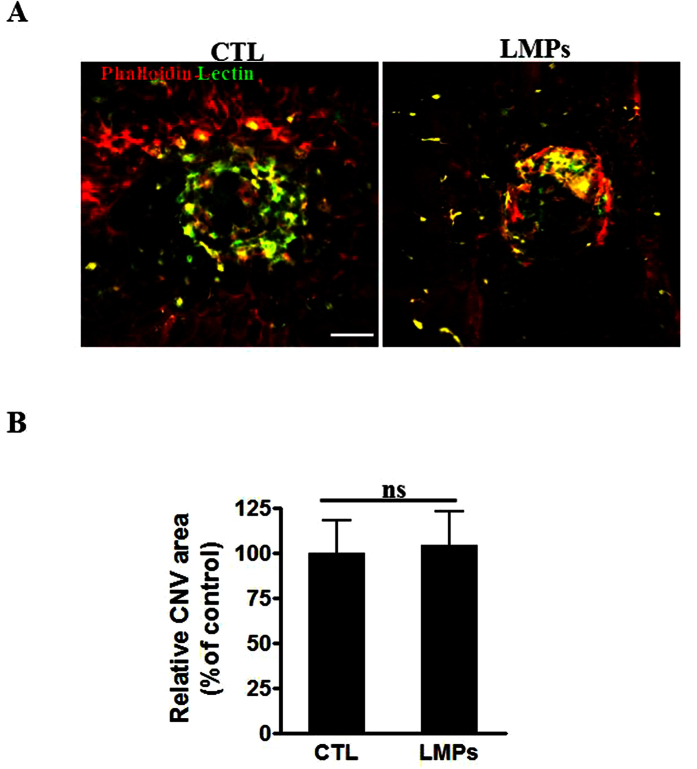
The antiangiogenic effect of LMPs was attenuated in CD36 knockout mice. (**A**) Representative images of choroidal flat-mounts of laser-induced CNV in CD36^−/−^ mice. The images were taken 7 days after the second LMPs intravitreal injection. Choroidal flat-mounts were immunostained with FITC-lectin (green) and phalloidin (red). Bar: 50 μm. (**B**) The CNV areas were quantified with computer-assisted semi-quantitative assay. Values were presented as percentage of control (set as 100%). Values are means ± SEM of 6 eyes (each eye containing 3 impact laser-induced CNV) for each condition. *P* > 0.05 between the two groups.

Since CD36 has been proven to be involved in the uptake of LMPs by macrophages, and LMPs are able to directly modulate macrophages gene expression, we surmised that the antiangiogenic effects of LMPs on laser-induced CNV could be significantly abolished in the CD36-deficient mice (CD36^−/−^ mice). As expected, no significant differences were observed between the areas of CNV of control CD36^−/−^ mice and LMPs-treated CD36^−/−^ mice (Fig. 7, *P* > 0.05). These results suggested that LMPs-mediated attenuation of CNV is dependent on CD36 expression. Therefore, it is most likely that both macrophages and endothelial cells play important roles in the LMPs-induced antiangiogenic effects. Further studies would be required to investigate the relative role of each cell type.

## Discussion

Macrophages are components of the immune system that play major roles in the pathological angiogenesis of the retina and the choroidal tissue^32^. In the present study, we report for the first time that lymphocytic microparticles are capable of altering the expression of key angiogenesis-related factors in macrophages, and that the scavenger receptor CD36 plays a crucial role in the mediation of these effects.

LMPs are membrane-derived vesicles derived from human apoptotic T cells^3^. We have demonstrated that LMPs can act as mediators of intercellular cross-talk and consequently induce a variety of cellular responses, including inhibition of proliferation of endothelial cells, and induction of apoptosis in airway epithelial and cancer cells^5^,^21^. Current study revealed that LMPs suppress cell proliferation of both mouse and human macrophages in a dose-dependent manner, and have no pro-apoptotic effects on these cells (Fig. 1 and Supplemental Figures S1 and S2). However, other studies indicate that LMPs derived from apoptotic Jurkat cells (human T lymphocyte) can induce macrophages to undergo apoptosis^33^. These divergent effects of LMPs could be due to the differences in their apoptotic stimuli, and/or in their parental cells.

Macrophages are immune cells with potential angiogenic functions. Emerging evidence suggests that macrophages have the ability to undergo two different activation pathways, classical and alternate ones, in response to different microenvironmental stimuli^31^. Classicallyactivated pro-inflammatory macrophages possessing an antiangiogenic phenotype are termed M1 macrophages^31^. Conversely, macrophages displaying a proangiogenic alternative phenotype are named M2^34^. M2 macrophages are capable of releasing angiogenic cytokines (e.g. VEGF, TNF-alpha, IL-10) and are known to contribute to wound healing and tissue repair as well as to pathological neovascularization^27^,^28^. Several markers have been used to identify M1 and M2 macrophages, such as the ratio of IL-10 and IL-12, CD206, CD80, CD86, and ARG^24^,^25^,^35^,^36^. LMPs treatment promoted differentiation of macrophages into M1 phenotype, evidenced by the increased expression of IL-12, CD80, CD86 and decreased expression of CD206 and ARG in LMPs-treated cells (Fig. 2). Similar results were obtained in human primary macrophages following a LMPs treatment (Supplemental Figures S3 and S4). Although the LMPs-treated macrophages exhibit M1 phenotype, they do not express more TNF-alpha, a major inflammatory factor (Supplemental Figure S5). Most interestingly, LMPs dramatically modulate the expression of a panel of angiogenesis-related factors in macrophages (Fig. 3B). The expression of key angiogenic factors such as proangiogenic factors, VEGFa and FGF, was decreased, while the expression of antiangiogenic factors, pigment epithelium-derived factor (PEDF) and TSP-1 was increased (Fig. 3C). These changes of expression of angiogenesis-related factors in LMP-treated macrophages had significant impact on endothelial cell proliferation as well as on CNV of choroidal explants from mouse and human (Fig. 4D and Supplemental Figure S6). Taken together, these results suggested that LMPs have a strong effect on modulating the angiogenic proprieties of macrophages. To investigate whether the antiangiogenic effect observed in this study is LMPs specific, we repeated the experiments of cell viability assay, FACS analysis of polarization of macrophages and *ex vivo* CNV assay using different controls such as endothelial cell derived microparticles (EMPs), and human T lymphocytes (CEM T cells, the parent cells of LMPs). These study revealed T lymphocytes have no effect on macrophages viability, and EMPs have no significant effect on macrophages polarization (data not shown). Moreover, opposite to the effect of LMPs, EMPs increased the retinal endothelial cell growth and exhibited pro-angiogenic effects in the *ex vivo* choroidal angiogenesis assay (Supplemental Figure S7). Nonetheless, without optimal control, we cannot exclude the possibility that microparticles derived from other apoptotic cells may be able to modulate macrophage function and possess antiangiogenic effects.

Previously, we have demonstrated that LMPs are taken up by target cells through receptor-mediated endocytosis or phagocytosis^5^,^37^. The reason of investigating of CD36 involvement in mediating the effect of LMPs on macrophages is due to the facts that CD36 is highly expressed in macrophages, and it plays an important role in angiogenesis^38^. Following observations supported our conclusion that CD36 does play a pivotal role in mediating the effects of LMPs: (1) CD36 colocalized with LMPs in macrophages (Fig. 4A); (2) blockade of CD36 prevented LMPs uptake by macrophages (Fig. 4B); (3) inhibition of CD36 in macrophages altered the effect of LMPs on the expression of angiogenesis-related factors (Fig. 4C); (4) blockade of CD36 in macrophages also attenuated the inhibitory effect of LMPs in choroidal angiogenesis assays (Fig. 4D); (5) the antiangiogenic effect of LMPs was completely abolished in CD36 KO mice (Fig. 7). Nonetheless, CD36 is a multifunctional membrane receptor which can act as an antiangiogenic or proangiogenic factor depending on the microenvironment and its ligands^18^,^39^,^40^,^41^. We reported previously that LMPs upregulated CD36 expression in immortalized human microvascular endothelial cells^4^, we also observed the upregulated expression of CD36 in LMPs-treated RAW 264.7 (Supplemental Figure S8). Thus, CD36 may mediate downstream events following LMPs ingestion and act as an antiangiogenic effector as well.

The mechanisms by which LMPs influence macrophage activity following their interaction with CD36 on the surface of macrophages have not been well defined. Whereas based on published reports^42^,^43^,^44^,^45^ and our observations, we surmised that there are two possible CD36-dependent signal transduction pathways may proceed in macrophages. Firstly, CD36 function as traditional signal transduction receptor initiating a signaling cascade conferred upon LMPs binding. This is because LMPs lose their membrane asymmetry and express anionic phospholipids, such as phosphatidylserine on their surface^42^. Thus LMPs become the endogenous CD36 ligands. The lipids of LMPs binding to CD36 mediates the activation of the C-Jun N-terminal kinase (JNKs). The JNKs belong to the conserved and ubiquitous signaling network of mitogen-activated protein kinases (MAPKs), and they are essential mediators of relevant pro-inflammatory functions in macrophages and microglia^46^. Particularly, JNK1 and JNK2 positively regulate IL-12 production in macrophages^47^,^48^. Relevantly, we observed that JNK1 expression was increased in LMPs-treated macrophages (Supplemental Figure S8), which indicated a possibility that LMPs activate the CD36-dependent MAP kinases JNKs and consequently induce IL-12 expression. Nonetheless, the function of JNKs in LMPs-induced antiangiogenic effect in macrophages need be further verified. Secondly, we contemplated that CD36 facilitates the internalization of bioactive components from LMPs that participate in the initiation of a transcriptional program that includes upregulation of its own gene as well as those of other critical genes involved in the function of macrophages. This is because we observed CD36 expression was significantly increased by LMPs treatment (Supplemental Figure S8). Moreover, we recently analyzed the microRNAs sequences in the LMPs, and found several abundant miRNAs possess potential regulatory effects on macrophages activity according to the reported^49^,^50^,^51^. The precise role of these miRNAs in LMPs remains to be determined. Taken together, in macrophages, LMPs might directly bind to CD36 and activate its downstream signaling resulted in an increasing expression of IL-12, the marker for M1 phenotype. Meanwhile, transfer the components of LMPs into macrophages may be mediated by CD36, and these active components consequently regulate genes expression including CD36, and the resulting cytokines production causes the antiangiogenic effect. In such a way, LMPs modulated the angiogenic properties of macrophages which may related to the suppression of CNV.

Neovascularization plays a key role in the pathophysiology of several diseases of the retina such as retinopathy of prematurity (ROP), diabetic retinopathy and choroidal neovascularization associated with age-related macular degeneration (AMD). LMPs possess strong antiangiogenic effect through direct targeting of vascular endothelial cells, and this effect was confirmed in *ex vivo* and *in vivo* ROP models^3^,^4^,^5^. As previously reported^6^, LMPs had no significant anti-angiogenic effect on mouse RPE-free choroidal angiogenesis (Fig. 4C), and the lack of anti-angiogenic effect of LMPs was also observed on human RPE-free choroidal explants (Supplemental Figure S6). The laser-induced murine model of CNV is widely used to mimic wet AMD by compromising Bruch’s membrane to induce CNV formation in the subretinal region, allowing visualization and evaluation of the morphologic changes of experimental CNV. It has been a valuable tool to study the diverse components of complex CNV lesions^52^. In line with the anti-angiogenic effects of LMPs observed in other pathological angiogenesis-related models^3^,^4^,^5^,^6^, intravitreal injection of LMPs significantly suppressed laser-induced CNV (Fig. 5). In this model, LMPs uptake by macrophages led to an increased IL-12 expression (Fig. 6) suggesting that LMPs are capable to reprogram macrophage functions *in vivo*. In addition, LMPs-induced expression changes of key angiogenic factors in the CNV areas may contribute to the overall CNV suppression.

In conclusion, LMPs promoted macrophages polarization toward antiangiogenic phenotype, and the resulting antiangiogenic activity of macrophages was confirmed on both murine and human choroidal explants as well as *in vivo* CNV murine model. Moreover, CD36 played a critical role in mediating the effect of LMPs on macrophages. This study highlighted the ability of LMPs to alter the gene expression pattern of angiogenesis-related factors in macrophages, and provided important information for a new therapeutic approach to efficiently interfere with both vascular and extravascular components of CNV.

## Methods

### LMPs production

Human CEM T cells were purchased from American Type Culture Collection (ATCC) and grown in X-VIVO medium (Cambrex). LMPs were generated as described previously^3^. Briefly, CEM T cells were treated with 0.5 μg/ml actinomycin D (Sigma-Aldrich) for 24 hours. A supernatant was obtained by centrifugation at 750 g for 15 min, then 1500 g for 5 min to remove cells and large debris. LMPs from the supernatant were washed after 3 centrifugation steps (50 min at 12,000 g) and recovered in PBS. Washing medium from the last supernatant was used as a control (CTL) unless otherwise noted. Up to now, there is no proper control for microparticles due to their heterogeneous components and unclarified pathophysiological roles. However, this last washing medium ensures that the effects seen are due to LMPs and not due to any soluble factor present in the preparation, which has been widely used as the control vehicle for each individual microparticle according to the published literatures^2^,^53^,^54^. LMPs were characterized with annexin V (BD bioscience) staining by fluorescence-activated cell sorting (FACS) analysis. The concentrations of LMPs were determined using the Bio-Rad protein assay. A lipophilic fluorescent stain Dil (1,1′-Dioctadecyl-3,3,3′,3′-Tetramethylindocarbocyanine Perchlorate) was added to CEM T cells 24 h before actinomycin D treatment. The process generated fluorescent Dil-labelled LMPs (Dil-LMPs).

### Cell culture

RAW 264.7 macrophages (murine cell line) were purchased from ATCC, and cultured in Dulbecco’s Modified Eagle’s Medium (Gibco, Life Technologies), supplemented with 10% FBS (Gibco, Life Technologies) and 1% penicillin/streptomycin.

### Proliferation and apoptosis assays

Macrophages (RAW 264.7) were incubated for 24 h with indicated concentrations of LMPs, then cell proliferation was evaluated by [^3^H]-thymidine incorporation assay. In co-culture experiments, human retinal epithelial cells (HREC) were seeded in the lower compartment of the Transwell unit (0.4 μm pore, Mississauga, ON) and macrophages or LMPs-pretreated macrophages (24 h pre-treatment of LMPs) were placed on the Transwell inserts. After 24 hours of co-incubation, HREC were assayed for proliferation rate. The relative cell proliferation rate is presented at a percentage of control (set as 100%). For apoptosis assay, the RAW 264.7 cells at approximately 60% confluence were treated with 10 or 20 μg/ml of LMPs for 24 hours followed by staining with reagents from a Vybrant apoptosis assay kit (Molecular Probes, Invitrogen, Carlsbad, CA) according to the manufacturer’s protocol. Apoptosis was determined by flow cytometry using FacsCalibur (BD Biosciences) and expressed as apoptotic rate (the percentage of apoptotic cells over the total number of cells). We observed that LMPs at 10 μg/ml concentration has no effect on macrophages viability, and used this LMPs dose in the subsequent *in vitro* studies.

### Quantitative RT-PCR and PCR array

Total RNA was extracted from RAW 264.7 cells and choroidal tissues using an RNA extraction kit (Qiagen, Mississauga, ON). DNase-treated RNA was then converted into cDNA using M-MLV reverse transcriptase (Invitrogen). Quantitative analysis of gene expression was performed on a Stratagene Mx3000p sequence detection system with SYBR Green Master Mix Kit (BioRad). Each sample was analyzed in triplicate and threshold cycle numbers were averaged. Gene expression was normalized to 18S, and the percentage of change was calculated according to a previously described formula^55^. PCR primers were synthesized by Alpha DNA (Montreal, Quebec, Canada) based on the following sequences: IL-10, forward 5′-TGGCCACACTTGAGAGCTGC-3′, reverse 5′-TTCAGGGATGAAGCGGCTGG-3′; IL-12, forward 5′-CAACATCAAGAGCAGTAGCAG-3′, reverse 5′-TACTCCCAGCTGACCTCCAC-3′; VEGFa, forward 5′-TGCAGGCTGCTGTAACGATG-3′, reverse 5′-GAACAAGGCTCACAGTGATTTTCT-3′; PEDF, forward 5′-TCGAAAGCAGCCCTGTGTT-3′, reverse 5′-AATCACCCGACTTCAGCAAGA-3′; FGF forward 5′-CAACCGGTACCTTGCTATGA-3′, reverse 5′-TCCGTGACCGGTAAGTATTG-3′, TSP-1, forward 5′-AACAAAGGACCTCCAAGCTATCTG-3′, reverse 5′-GGGAGGCCGCTTCAGC-3′; 18S rRNA, forward 5′-CCTGCGGCTTTAAATTT GACTCA-3′, reverse ′-GCTATCAATCTGTCAATCCTGTC-3′.

For the SABioscience Mouse Angiogenesis array (PAMM-024), total RNAs from cells were isolated according to manufacturer’s instructions by using RNeasy Microarray Tissue Mini Kit (SABioscience, Qiagen). 1 μg of RNA was used for RT-PCR array which was carried out on Stratagene model Mx3000p with SYBR Green (SABiosciences). The data was analyzed using the RT2 Profiler PCR array data analysis software (SABiosciences).

### Flow cytometry analysis

For intracellular staining of IL-10, IL-12, and Arginase-1 (ARG), cells were treated with Brefeldin A and permeabilized using the Fixation & Permeabilization kit (eBioscience) according to the recommended protocols. The treated cells were then incubated with following first antibodies against mouse IL-10-APC (JES5-16E3), IL-12-PE (C17.8, eBioscience), Arginase-1 (PA5-22009; Fisher Scientific) respectively, followed by staining with secondary antibody of goat anti-rabbit IgG-FITC (Santa Cruz). For extracellular staining of the macrophage receptors, cells were incubated with the following antibodies: anti-mouse CD206-APC (C068C2, Biolegend), CD86-PE (GL1), CD80-PE (16-10A1, eBioscience) for 30 min on ice and washed three times with PBS containing 0.05% bovine serum albumin. Cells were re-suspended in 2% paraformaldehyde and then subjected to flow cytometry analysis using FacsCalibur (BD Biosciences).

### Detection of CD36 expression and macrophages uptake of LMPs

RAW 264.7 were seeded onto coverslip in 12 well plates. Next day, cells were incubated with 10 μg/ml Dil-LMPs for 4 h. After LMPs treatment, macrophages were fixed and nuclei were stained with DAPI (1:5000; Invitrogen). The expression of CD36 was detected with anti-CD36-FITC (1:100, Serotec, Oxford, UK). Images were taken using a laser scanning confocal microscope (Zeiss LSM 510). To investigate whether CD36 is involved in the uptake of LMPs, macrophages were seeded into 96-well plates, and pretreated with anti-CD36 antibodies (15 μg/mL) to block CD36 receptors (Cayman Chemical, Ann Arbor, MI) or with isotype-matched control antibodies (Sigma) for 3 hours followed by 4 hours DiI-LMPs incubation. The DiI fluorescence intensity was measured using spectrofluorometer and presented as mean fluorescence intensity (MFI).

### Animals

C57BL/6 J and CD36 knockout mice (CD36 KO) were purchased from the Jackson Laboratory (Bar Harbor, Maine, USA). For the CNV model, mice were anesthetized using intraperitoneal administration of ketamine hydrochloride/xylazine. Their pupils were dilated with 1% tropicamide. A coverslip was placed over the cornea, and Argon laser photocoagulation (400 mW 0.05 s) was performed to rupture Bruch’s membrane at four locations in each eye^56^,^57^. Mice were randomly grouped to receive intravitreal injections on day 1 and day 7 with the last wash-treated from LMPs (control group), LMPs (50 μg) (treatment group) or Dil-LMPs (50 μg). The 50 μg of LMPs is the optimal dose seen in our previous *in vivo* and *ex vivo* experiments^5^. Mice were sacrificed 14 days after the laser photocoagulation, and the eyes were rapidly enucleated. The choroids were prepared for immunofluorescence and RT-PCR analysis. All procedures in this study were approved by the Maisonneuve-Rosemont Hospital Animal Welfare Committee, and are in accordance with the guidelines of the Canadian Council on Animal Care and the Guide for the Care and Use of Laboratory Animals published by the US National Institutes of Health.

### Preparation of retinal pigment epithelium (RPE)-free choroidal explants

The RPE-removed choroidal explants were prepared according to a previously described procedure^6^. Mice eyes were dissected in a Petri dish containing 1X Hank’s balanced Salt Solution (HBSS, Invitrogen). The blood vessels, connective and fatty tissues were removed from the exterior of the eyeballs. The eyes were then incubated at 37 °C for 45 min with 2% diapase II (neutral protease; Roche) in HBSS. After removing the anterior segment, retina and sclera, the RPE layer was peeled off from the choroid with fine forceps. The choroid without RPE was sliced into 1~2-mm sections and placed in growth factor–reduced basement membrane matrix. The effectiveness of the RPE removal was histologically evaluated^6^.

### Treatment of choroidal explants and measurement of neovascularization

Choroidal explants were cultured at 37 °C in 5% CO_2_ for 4 days. The culture medium was changed on day 5 and explants were incubated with saline, 50 μg/mL of LMPs or co-cultured with LMPs-pretreated macrophages, CD36 antibody pre-treated macrophages, or CD36 pre-treated + LMPs treated macrophages in a Transwell for 48 hours. For the pre-treatments, macrophages were pre-incubated with CD36 specific antibody 45 μg/mL for 3 hours, or/and 24 hours of LMPs treatment. Photographs of individual explants were taken before and after the treatment using an Axiovert 200 M inverted microscope (Zeiss). The neovessel areas were determined using Image-Pro Plus software.

### Immunohistochemistry **s**taining of choroidal flat-mounts

The choroidal flat-mounts were prepared as described previously^58^. In brief, the eyes of mice that received an intravitreal injection of control vesicles, LMPs, or DiI-LMPs (n ≥ 3 for each group) were enucleated and fixed in 4% PFA. After the cornea, lens and retina was removed, the choroid-sclera was permeabilized in 1.0% Triton X100 and blocked in 10% normal goat serum. A first antibody against the ionized calcium-binding adapter molecule 1 (Iba1) (1:500; Wako Chemicals, USA) and second antibodies the Alexa-448 -conjugated goat anti-rabbit or Alexa-594 conjugated goat anti-rabbit (1:1000; Invitrogen) were used to specifically identify macrophages. FITC-conjugated lectin was used to stain endothelial cells (1:100; Sigma-Aldrich, St. Louis, MO); Rhodamine Phalloidin (R415; 1:400; Santa Cruz Biotechnology, Santa Cruz, CA) was used for RPE cells. Antibody against mouse IL-10-APC (1:1000) and against mouse IL-12-PE clone (1:1000) were used to detect IL-10 and IL-12, respectively. Cell nuclei were stained with DAPI (1:5000; Invitrogen). Labeled flat-mounts were examined with a laser scanning confocal microscope (Zeiss LSM 510).

### Laser-capture microdissection

The mice eyes were snap frozen in optimal cutting temperature compound (OCT, Fisher). Sections of 16 μm were dissected with a Zeiss Observer microscope equipped with Palm MicroBeam device for laser-capture microdissection. Total RNAs were extracted from these dissected tissues as described^59^.

### Statistical analysis

All experiments were performed in duplicate or triplicate and repeated independently at least 3 times. Values are presented as means ± SEM. Data were analyzed by one-way ANOVA followed by post-hoc Bonferroni tests for comparison of means. Statistical significance was set at *P* < 0.05.

## Additional Information

**How to cite this article**: Tahiri, H. *et al*. Lymphocytic Microparticles Modulate Angiogenic Properties of Macrophages in Laser-induced Choroidal Neovascularization. *Sci. Rep*. **6**, 37391; doi: 10.1038/srep37391 (2016).

**Publisher’s note:** Springer Nature remains neutral with regard to jurisdictional claims in published maps and institutional affiliations.

## Supplementary Material

Supplementary Information
