# Supplementary material for: Lymphocytic Microparticles Modulate Angiogenic Properties of Macrophages in Laser-induced Choroidal Neovascularization
Source: Sci Rep. 2016 Nov 22;6:37391. doi: 10.1038/srep37391 (PMC5118818; doi:10.1038/srep37391)
Supplement: Supplementary Information [file srep37391-s1.pdf]

Supplementary information

Lymphocytic Microparticles Modulate Angiogenic Properties of Macrophages in Laser-induced Choroidal Neovascularization

Houda Tahiri[1,3], Samy Omri[2], Chun Yang[3], François Duhamel[1], Suzanne Samarani[4], Ali Ahmad[4], Mark Vezina[5], Martin Bussières[6], Elvire Vaucher[7], Przemyslaw Sapieha[2,8], Gilles Hickson[9], Karim Hammamji[8], Réjean Lapointe[10], Francis Rodier[10,11], Sophie Tremblay[13,14], Isabelle Royal[10], Jean-François Cailhier[10], Sylvain Chemtob[1,2,3,8,12], and Pierre Hardy[1,3,12] *

[1]Department of Pharmacology, Université de Montréal, Montréal, QC, Canada; [2]Research Center Hôpital Maisonneuve-Rosemont, Université de Montréal, Montréal, QC, Canada; [3]Research Center CHU Sainte-Justine, Université de Montréal, Montréal, QC, Canada; [4]Departments of Microbiology and Immunology, Université de Montréal, Montréal, QC, Canada; [5]Charles River Laboratories, Senneville, Montreal, QC, Canada ; [6]V&O Services, Saint-Lazare, QC, Canada ; [7]School of Optometry, Université de Montréal, Montréal, QC, Canada; [8]Department of Opthalmology, Université de Montréal, Montréal, QC, Canada; [9]Department of Pathology and Cell Biology, Université de Montréal, Montréal, QC, Canada; [10]Institut du Cancer de Montréal, CRCHUM – Centre de Recherche du Centre Hospitalier de l'Université de Montréal and Department of Medicine, Université de Montréal, Montréal, QC, Canada; [11]Department of Radiology, Radio-Oncology and Nuclear Medicine, Université de Montréal, Montréal, QC, Canada; [12]Department of Pediatrics, Université de Montréal, Montréal, QC, Canada; [13] University of British Columbia, Vancouver, BC, Canada, [14]Centre for Molecular Medicine and Therapeutics, Vancouver, BC, Canada

Corresponding author:

Pierre Hardy, M.D. Ph.D.

Research Center of CHU Sainte-Justine, 3175 Côte-Sainte-Catherine, Room 2714

Montréal, Québec, H3T 1C5

Phone: (514) 345-4931 (ext. 3656), Fax: (514) 345-4801

E-mail: pierre.hardy@recherche-ste-justine.qc.ca

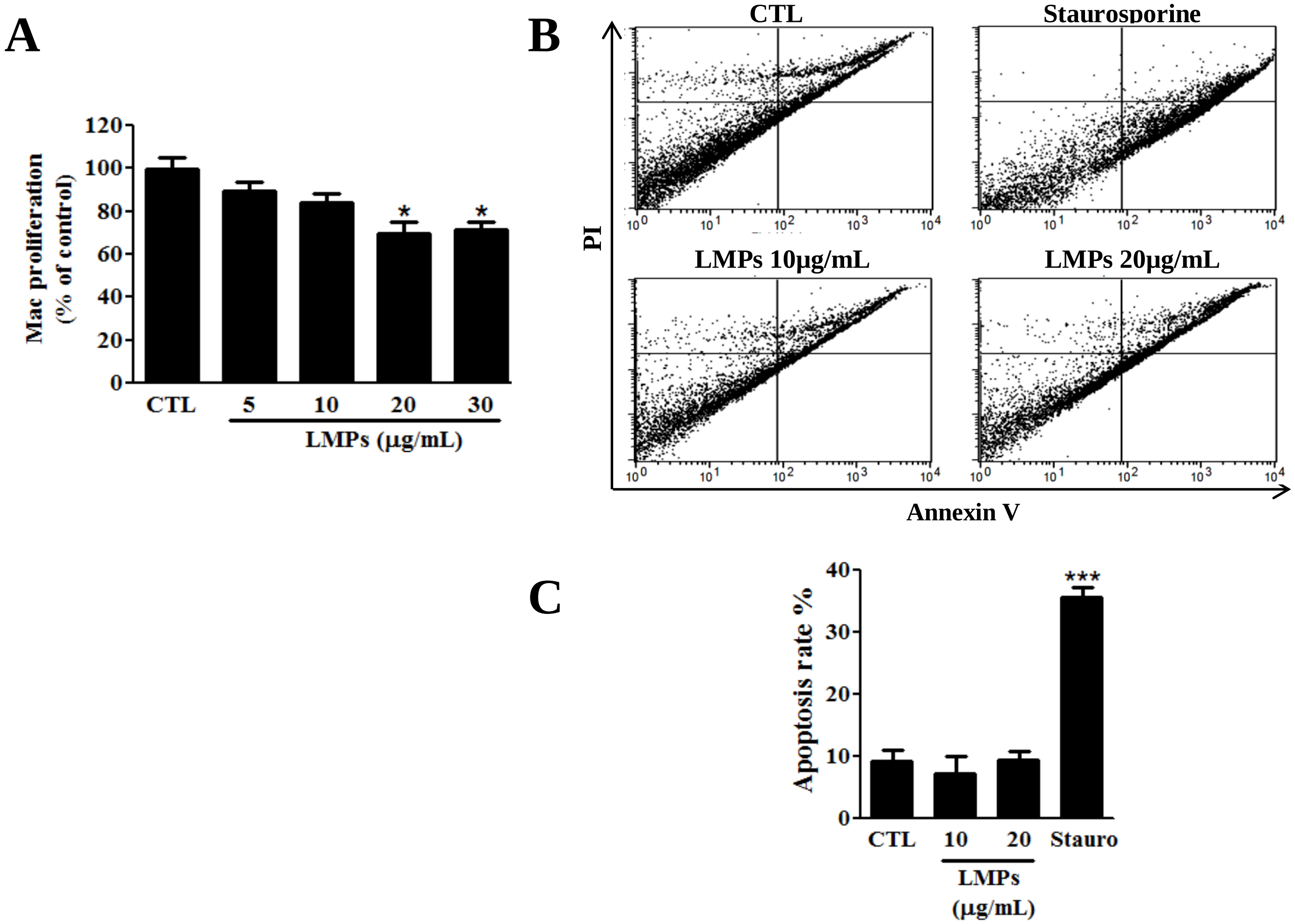

Figure S1: LMPs dose-dependently inhibited cell proliferation of bone marrow-derived macrophages (BMDM). With the techniques support from Dr. Jean-François Cailhier (University of Montreal), we isolated the bone marrow-derived macrophages (BMDM) from femurs of C57BL/6 mice by standard sterile techniques. BMDM were matured for 7 days in Dulbecco's modified Eagle's medium (Wisent Inc., St-Bruno, Quecbec, Canada) with 10% FBS (Wisent Inc.), penicillin/streptomycin (100 mg/ml) (Wisent Inc.), and 20% L929 cell-conditioned medium as a source of macrophage-colony stimulating factor.

(**A**) Indicated concentrations of LMPs were incubated with BMDM for 24 hours. The proliferation of macrophages was determined using ^{3}H-thymidine incorporation and values were presented as a percentage of control (CTL). *P < 0.05 vs. CTL. (**B**) Representative results of flow cytometry analysis of BMDM cell apoptosis after 24-hour treatment with indicated concentrations of LMPs, or staurosporine (positive control). FACS analysis was performed after macrophages were stained with Annexin-FITC and propidium iodide using Vybrant Apoptosis assay kit. Note that the staurosporin-treated cells undergo apoptosis and become PI+/Annexin V+ in the later stages of apoptosis. **(C)** The apoptosis rates were presented as the percentage of apoptotic cells relative to the total cell numbers. Values are means ± SD of 3 individual experiments. ***P < 0.001 vs CTL. Mac and Stauro indicate macrophages (BMDM) and staurosporin, respectively. CTL (control) means the cells treated with the last wash from the LMPs.

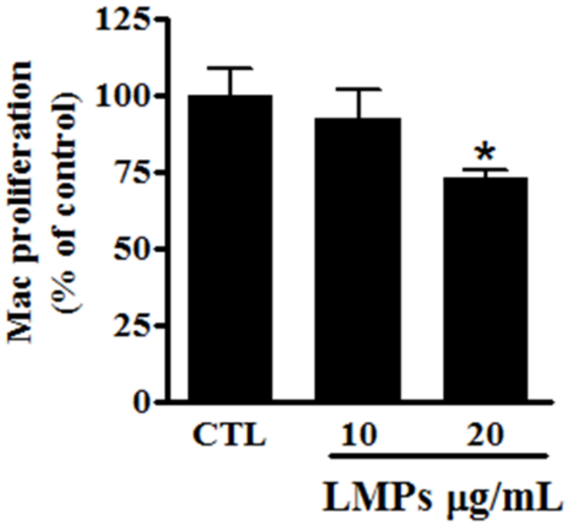

Figure S2: LMPs dose-dependently inhibited human macrophages proliferation (HL60). Indicated concentrations of LMPs were incubated with human macrophages cell line (HL60) for 24 h. The cell proliferation was determined using ^{3}H-thymidine incorporation assay and values were presented as percentage of control. *P<0.05 vs. CTL

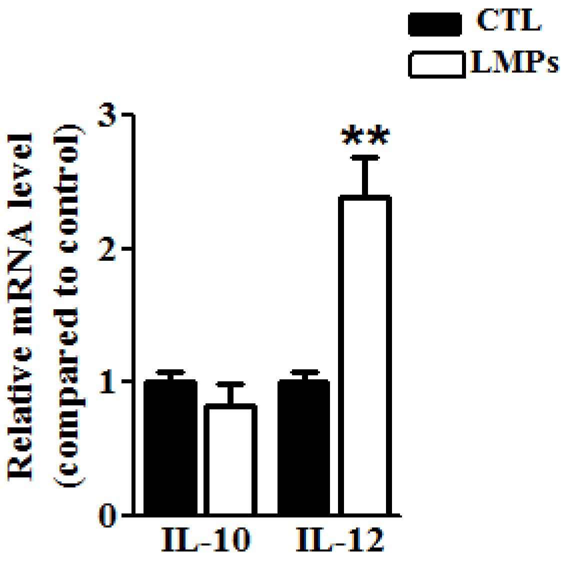

Figure S3: LMPs altered the expression of IL-10, IL-12 in human macrophages. With the techniques support from Dr. Ali Ahmad (Sainte-Justine Hospital, Universaity of Montreal), we isolated the human peripheral blood mononuclear cells (PBMCs) using centrifugation of blood over Ficoll-Hypaque (Pharmacia, Montreal, QC) and washed with the culture medium without FCS and antibiotics. Human monocytes were isolated from PBMCs by negative selection using a Human Monocyte Enrichment Kit (StemSep; Stem Cell Technology, Vancouver, BC), and then differentiated into macrophages M2 in culture medium containing 10% FCS, 5% human AB serum, and 20 ng/ml of recombinant human granulocyte-macrophage colony-stimulating factor (M-CSF; BioSource, Camarillo, CA). The mRNA expression levels of IL-10 and IL-12 were quantified by quantitative RT-PCR after macrophages were treated with 10μg/mL of LMPs for 24 hours. The values were presented as fold change relative to the control set as 1. ***P* < 0.01 vs. CTL.

The sequences of PCR primers used in RT-PCR:

human IL-10: forward 5'-GCCTAACATGCTTCGAGATC-3’,
reverse 5’-TGATGTCTGGGTCTTGGTTC-3‘;

human IL-12: forward 5'-GCCTACCCATTGAAGTCGTG-3',
reverse 5'-GGTTTGATGATGTCCC TCATG-3’.

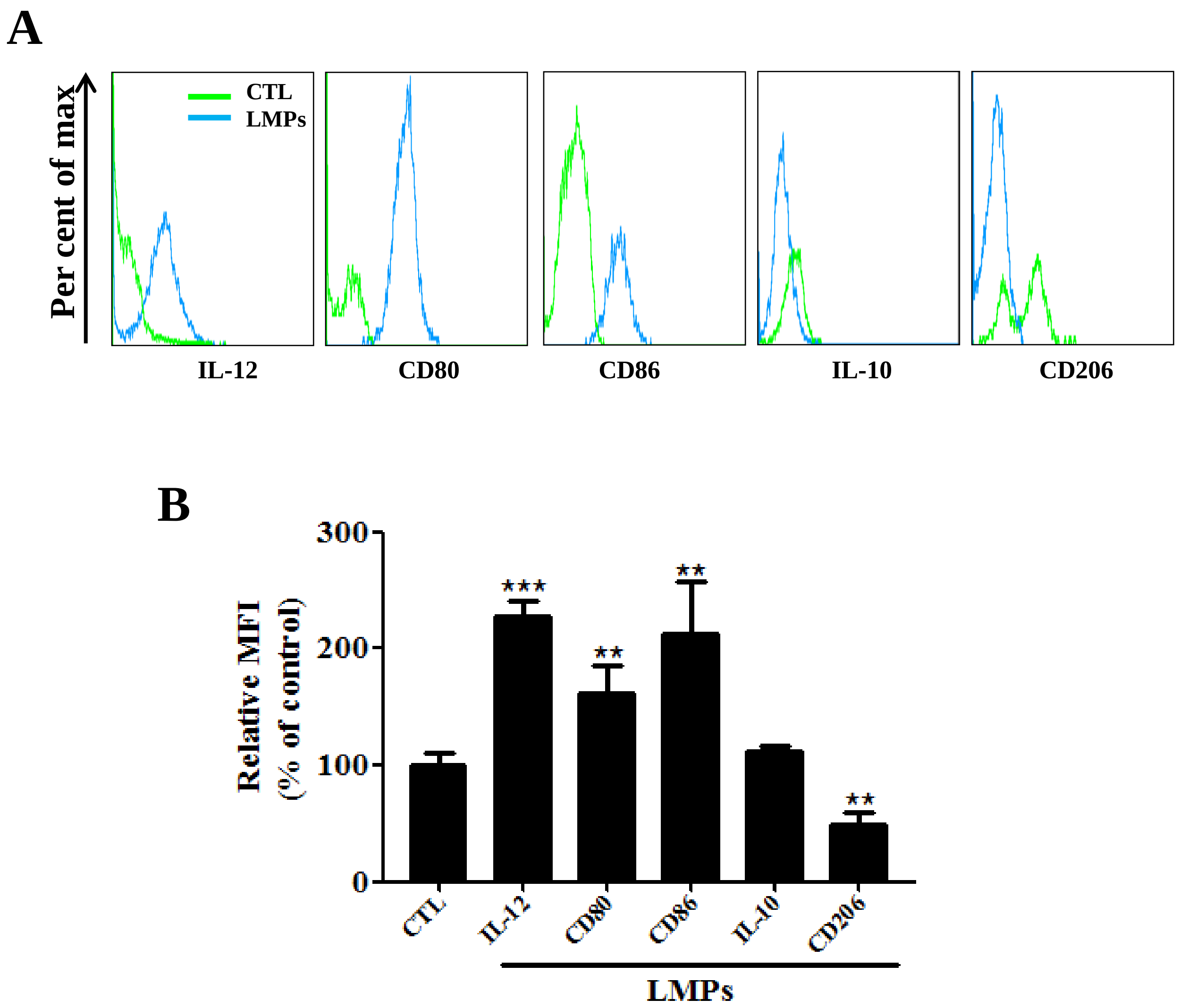

Figure S4: LMPs altered the expression of M1 and M2 markers in human macrophages. (**A**) Representative FACS analysis of the expression of IL-12, CD80, CD86, IL-10, and CD206 in human macrophages after 24-hour treatment of 10µg/mL of LMPs. (**B**) The number of cells expressing of IL-12, CD80, CD86, IL-10 and CD206 were calculated respectively and presented as a percentage of control (set as 100%). Values are means ± SEM of 3 individual experiments. ***P<0.001, **P<0.01 vs. CTL.

For intracellular staining of IL-10 and IL-12 cells, following specific antibodies were used: human IL-10-PE (clone, JES3-9D7), mouse IL-12-Alexa Fluor 647 (SNKY35) (eBioscience). For extracellular staining, macrophages were incubated with the followed antibodies anti-mouse CD206-PE (c19.2) (Biolegend),mouse CD86 PE (GL1), Mouse CD80 APC (2D10) (eBioscience).

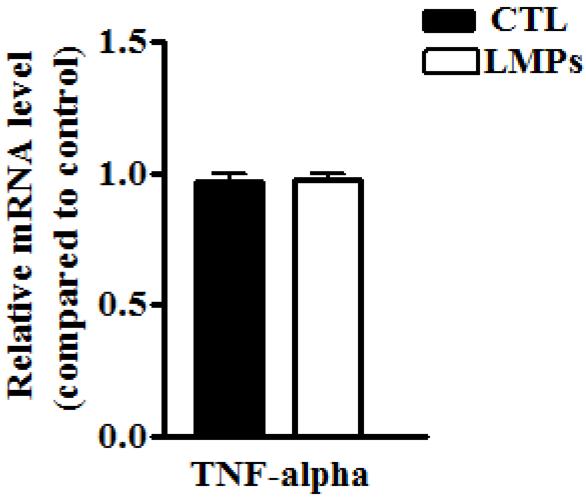

Figure S5: The expression of TNF-alpha in macrophages was not significantly affected by LMPs treatment. The mRNA expression level of TNF-alpha was quantified by quantitative RT-PCR after macrophages were treated with 10μg/mL of LMPs for 24 h. Values were presented as fold changes relative to control set as 1. $P > 0.05$ vs. CTL.

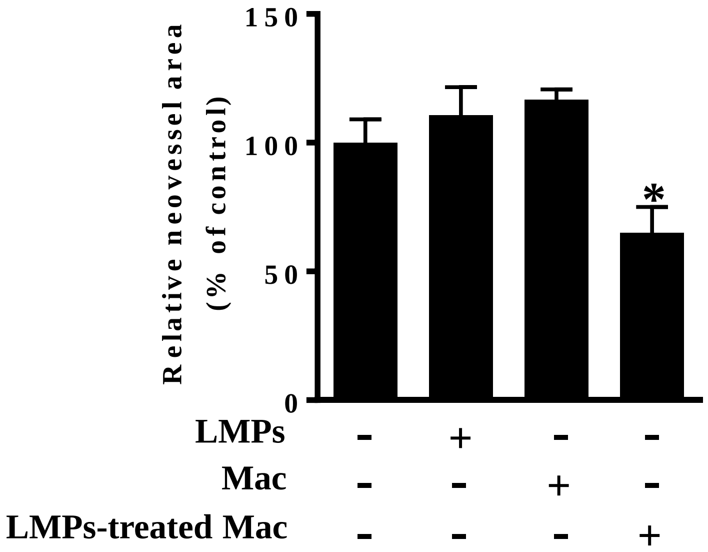

Figure S6: LMPs stimulated the antiangiogenic activity of human macrophages. In *ex vivo* angiogenesis assay, human RPE-free choroidal explants were cultured for 48h with normal medium (control), 50µg/mL of LMPs, co-cultured with macrophages (Mac), or LMPs-treated Mac. The neovessel areas were calculated and presented as percentage of control (set as 100%). *P <0.05 vs. CTL.

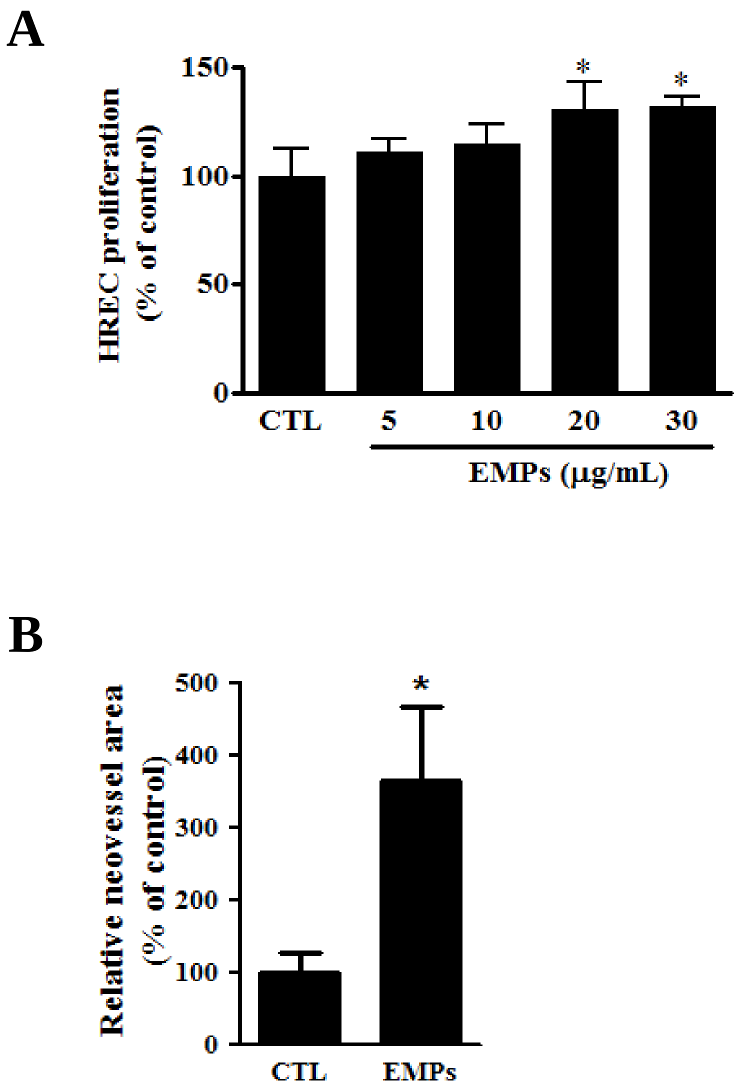

Figure S7: Microparticles derived from human endothelial cells (EMPs) exhibited pro-angiogenic effect. (A) Indicated concentrations of EMPs were incubated with HREC for 24 hours. The proliferation of HREC was determined using ^{3}H-thymidine incorporation and values are presented as a percentage of CTL. *$P < 0.05$ vs. CTL. (**B**) In *ex vivo* angiogenesis assay, mouse RPE-free choroidal explants were cultured for 48h with normal medium (control), 50µg/mL of EMPs. The neovessel areas were calculated and presented as percentage of control (set as 100%). *$P < 0.05$ vs. CTL.

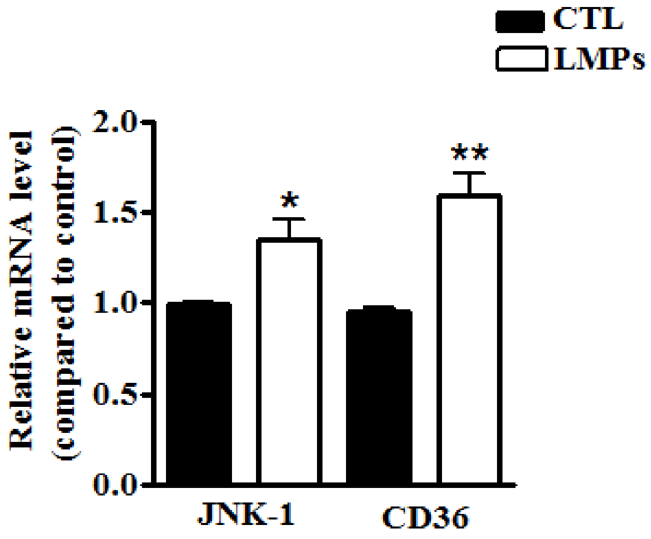

Figure S8: LMPs increased the expression of JNK-1 and CD36 in macrophages (RAW246.7). The mRNA levels were quantified by quantitative RT-PCR after macrophages were treated with 10μg/mL of LMPs for 24 h. Values were presented as fold changes relative to control group set as 1. **P* <0.05, ***P* <0.01 vs. CTL.

Gene	Fold change	Gene	Fold change	Gene	Fold change	Gene	Fold change	Gene	Fold change	Gene	Fold change
Akt1	1,11	Ang	0,39	Angpt1	1,90	Angpt2	4,50	Anpep	0,38	Bai1	1,60
Ctgf	1,24	Cxcl1	3,01	Cxcl2	4,69	Cxcl5	1,94	Edn1	3,34	Efna1	0,91
F2	1,22	F3	0,28	Fgf1	0,08	Fgf2	0,55	Fgf6	0,27	Fgfr3	0,65
Igf1	0,83	I1b	1,14	Il6	2.08	Itgav	0,84	Itgb3	0,07	Jag1	1,69
Mmp19	0,70	Mmp2	1,03	Mmp9	0,56	Nos	0,36	Nrp1	0,14	Nrp2	0,31
Prk2	2,20	S1pr1	0,02	Serpine1	0,09	Serpinf1	0,27	Smad5	0,18	Sphk1	0,35
Tgfbr1	0,18	Thbs1	1,60	Thbs2	1,07	Tie1	2,04	Timp1	1,08	Tomp2	0,26
Ccl11	1,09	Ccl2	14,33	Cdh5	0,99	Col18a1	2,21	Col4a3	1,42	Csf3	4,89
Efnb2	0,95	Egf	0,03	Eng	0,46	Epas1	2,77	Ephb4	0,11	Erbb2	1,34
Figf	1,52	Flt1	1,06	Fn1	0,87	Hgf	0,97	Hif1a	0.97	Ifng	1,42
Kdr	0.90	Lect1	0,72	Lep	0,04	Mapk14	2,31	Mdk	0,92	Mmp14	2,12
Pdgfa	1,02	Pecam1	0,59	Pgf	0,21	Plau	1,41	Pfg	0,36	Pfgs1	0,03
Tbx1	0,73	Tek	0,03	Tgfa	0,11	Tgfb1	0,08	Tgfb2	0,38	Tgfb3	2,08
Tnf	1,02	Tnfsf12	1,60	Tymp	0,75	Vegfa	0,06	Vegfb	0,77	Vegfc	0,08

Table S1: LMPs modulated the expression of angiogenesis-related genes in macrophages. RAW 246.7 cells were treated with 10µg/mL of LMPs for 24 hours, total RNAs were isolated and subjected to RT^2PCR Array analysis using a mouse angiogenesis array (PAMM-024) kit from SABioscience. The values were presented as fold changes relative to control genes set as 1.
